# Apolipoprotein A5 ameliorates MCT induced pulmonary hypertension by inhibiting ER stress in a GRP78 dependent mechanism

**DOI:** 10.1186/s12944-022-01680-4

**Published:** 2022-08-08

**Authors:** Jingyuan Chen, Jun Luo, Haihua Qiu, Yi Tang, Xiaojie Yang, Yusi Chen, Zilu Li, Jiang Li

**Affiliations:** 1 Department of Cardiovascular Medicine, Second Xiangya Hospital of Central South University, No. 139 Middle Renmin Road, Furong District, Changsha City, Hunan Province 410011 China; 2 Department of Cardiology, Clinical Medicine Research Center of Heart Failure of Hunan Province, Hunan Provincial People’s Hospital, The First Affiliated Hospital of Hunan Normal University, Hunan Normal University, Changsha, Hunan China

**Keywords:** Pulmonary arterial hypertension, Apolipoprotein A5, Endoplasmic reticulum stress, Glucose- regulated protein 78

## Abstract

**Background:**

Pulmonary arterial hypertension (PAH) is a chronic, progressive lung vascular disease accompanied by elevated pulmonary vascular pressure and resistance, and it is characterized by increased pulmonary artery smooth muscle cell (PASMC) proliferation. Apolipoprotein A5 (ApoA5) improves monocrotaline (MCT)-induced PAH and right heart failure; however, the underlying mechanism remains unknown. Here we speculate that ApoA5 has a protective effect in pulmonary vessels and aim to evaluate the mechanism.

**Methods:**

ApoA5 is overexpressed in an MCT-induced PAH animal model and platelet-derived growth factor (PDGF)-BB-induced proliferating PASMCs. Lung vasculature remodeling was measured by immunostaining, and PASMC proliferation was determined by cell counting kit‐8 and 5‐ethynyl‐2'‐deoxyuridine5‐ethynyl‐2'‐deoxyuridine incorporation assays. Coimmunoprecipitation-mass spectrometry was used to investigate the probable mechanism. Next, its role and mechanism were further verified by knockdown studies.

**Results:**

ApoA5 level was decreased in MCT-induced PAH lung as well as PASMCs. Overexpression of ApoA5 could help to inhibit the remodeling of pulmonary artery smooth muscle. ApoA5 could inhibit PDGF-BB-induced PASMC proliferation and endoplasmic reticulum stress by increasing the expression of glucose-regulated protein 78 (GRP78). After knocking down GRP78, the protecting effects of ApoA5 have been blocked.

**Conclusion:**

ApoA5 ameliorates MCT-induced PAH by inhibiting endoplasmic reticulum stress in a GRP78 dependent mechanism.

**Supplementary Information:**

The online version contains supplementary material available at 10.1186/s12944-022-01680-4.

## Introduction

Pulmonary arterial hypertension (PAH) is a chronic, progressive lung vascular disease accompanied by elevated pressure and resistance associated with endothelial dysfunction, abnormal pulmonary artery contraction, and vascular remodeling. If untreated or not well-controlled, PAH eventually develops into right heart failure and premature death [1]. Pulmonary artery smooth muscle proliferation and obstruction are among the most important features of PAH. Progressive remodeling and muscularization of small distal pulmonary arteries provide increased resistance [2, 3], and in PAH, this remodeling is characterized by high pulmonary artery smooth muscle cell (PASMC) proliferation and migration and an apoptosis-resistant phenotype [4].

In recent decades, although huge efforts have been made, clinical approach for PAH is focused on drugs that target vascular tone. An effective method of reversing the progression of this disease is not available, and current treatment strategies mainly provide symptom relief [5]. Thus, in the search for new therapeutic targets, new molecular or signaling pathways focused on pulmonary artery remodeling and PASMC proliferation are urgently needed [6].

Apolipoprotein A5 (ApoA5) is a lipoprotein that functions in the circulation as an important regulator of serum triglycerides (TGs) [7]. Several studies have proven that ApoA5 can be absorbed by extrahepatic tissues, such as adipose tissue and the heart, and functions as an important intracellular TG regulator [8, 9]. Previously, our group found that ApoA5 protected against obesity-related heart failure by inhibiting heart lipid deposition and free fatty acid uptake [10]. Meanwhile, dysregulated fatty acid metabolism and TG deposition showed destructive effects in the right ventricle (RV) of PAH patients and experimental models [11]. Decreased ApoA5 has been reported in the monocrotaline (MCT)-induced PAH animal model, while overexpressed ApoA5 could help relieve increased pulmonary pressure and right heart fibrosis [12]. However, none of these protective effects were exerted by modulating TG metabolism.

Although several researchers have reported that ApoA5 gene polymorphisms are closely related to vascular diseases like hypertension, coronary artery disease, and stroke, the fundamental underlying mechanism has not been investigated [13]. Recent studies indicated that ApoA5 may have a protective effect in acute inflammation and lipopolysaccharide (LPS)-induced fulminant liver failure and may represent a diagnostic and prognostic predictor in pediatric patients with sepsis [14, 15]. These studies demonstrated that beyond TG modulation, ApoA5 might have alternative effects on vascular diseases.

This study revealed that beyond its function in the right heart, ApoA5 could also be taken up by PASMCs and protect against MCT-induced PAH. Further in vitro studies were performed to demonstrated the underlying mechanism. ApoA5 overexpression inhibited PASMC proliferation by ameliorating endoplasmic reticulum (ER) stress through a glucose-regulated protein (GRP78) dependent mechanism.

## Methods

### Animals

Male Sprague‐Dawley (SD) rats around 6-week were purchased from SJA laboratory animal company (Changsha, China) and raised at the Central South University Experimental Animal Center following guidelines for experimental animal. 3 groups were randomly distributed: MCT + GFP group and MCT + APOA5 group, in which 60 mg/kg MCT was injected on Day 1 and green fluorescent protein (GFP) or ApoA5-overexpressing adenovirus was injected on Days 7 and 14, respectively; and Control group, in which the same amount of saline was administered through intraperitoneal and tail vein injections at the same times listed for the previous two groups.

### Cells

Primary PASMCs were isolated from rat pulmonary arteries by the substrate-attached explant method. SD rats weighing less than 200 g were used for cell isolation. Animals were euthanized with excessive CO_2_. After 5 min and certification of death, the chest was opened and all tissues surrounding the pulmonary artery were removed. The whole pulmonary artery and branches were moved to another plate. The inner layers were scraped to clean the endothelium and then cut into small pieces and placed at the bottom of the culturing flask. The morphologic appearance and immunofluorescence were determined with an anti‐α‐smooth muscle actin (α-SMA) antibody to characterize the PASMCs. Cells from passages 2–4 were used for the experiments.

### Cellular treatment, reagents, and transfection

GFP or ApoA5 adenovirus at a multiplicity of infection 5 was used for transfection. After 6 h, the medium was replaced with fetal bovine serum -free Dulbecco’s Modified Eagle’s media. Subsequently, 10 ng/ml platelet-derived growth factor (PDGF)-BB was added for an additional 24‐48 h.

For GRP78 siRNA transfection, scramble or GRP78 siRNA (purchased from RiboBio, Guangzhou, China) were used prior to adenovirus transfection. siRNA (10 nM) was incubated with Opti-MEM (Gibco, Carlsbad, CA, USA) and Lipofectamine RNAiMAX reagent (Life Technologies, Gaithersburg, MD, USA) at room temperature for 5 min before transfection, which continued for approximately 6 h.

### Cell proliferation assay

Cell Counting Kit‐8 (CCK‐8) assay (Dojindo, Kumamoto, Japan) and 5‐ethynyl‐2'‐deoxyuridine (EdU) staining (Beyotime, Shanghai, China) were used. After all treatments, CCK‐8 solution (10 µl) or 10 µM EdU was added. For the CCK-8 assay, the absorbance was then measured at 450 nm (Thermo, MA, USA). For the EdU incorporation assay, cells were washed with PBS and fixed with 4% paraformaldehyde after incubation. All steps followed the manufacturer’s instructions.

### Coimmunoprecipitation (co-IP), mass spectrometry (MS), and bioinformatic analysis

Co-IP was detected using a Co-Immunoprecipitation Kit (Thermo, MA, USA). After harvesting by IP lysis/wash buffer and concentration measurement, 1000 μg protein per sample was used for the reaction and precleared by control agarose resin. ApoA5 antibodies were coupled to agarose resin following the manufacturer's instructions. Resin and protein were incubated at 4 ℃ overnight, and then the proteins were washed off and harvested. After running a gel and performing verification by Coomassie brilliant blue staining, the products were sent for MS analysis.

MS analysis and functional annotation and classification were conducted by PTM Bio (Zhejiang, China). Functional categorization and pathway construction were performed using R software.

### Western blotting

Tissue and cells were lysed in NP-40 buffer that contained protease and phosphatase inhibitors. β-Tubulin was used as a loading control. Antibodies against ApoA5 were purchased from Santa Cruz Biotechnology (CA, USA), and GRP78 and α-SMA were obtained from Abcam (Cambridge, USA). Double-stranded RNA-activated protein kinase (PKR)-like endoplasmic reticulum kinase (PERK), proliferating cell nuclear antigen (PCNA), and phospho-PERK were from Cell Signaling Technology (MA, USA). Activating transcription factor 6 (ATF6) antibody was from ABclonal (ABclonal Technology, Wuhan, China). Inositol requiring 1 alpha (IRE1α), and phospho-IRE1α were purchased from Novus (Littleton, CO, USA).

### Statistical analysis

Statistical analyses were performed using SPSS 22.0 (IBM Corp, NY, USA). *P* < 0.05 was considered statistically significant. Differences between the 2 groups were compared by independent t tests. One-way analysis of variance was conducted for differences for more than 3 groups. For post hoc analysis, the LSD comparison method was used if homogeneity of variance was observed. Otherwise, Dunnett’s T comparison was performed.

## Result

### Decreased ApoA5 synthesis and uptake in the PAH animal model

**Fig. 1:**
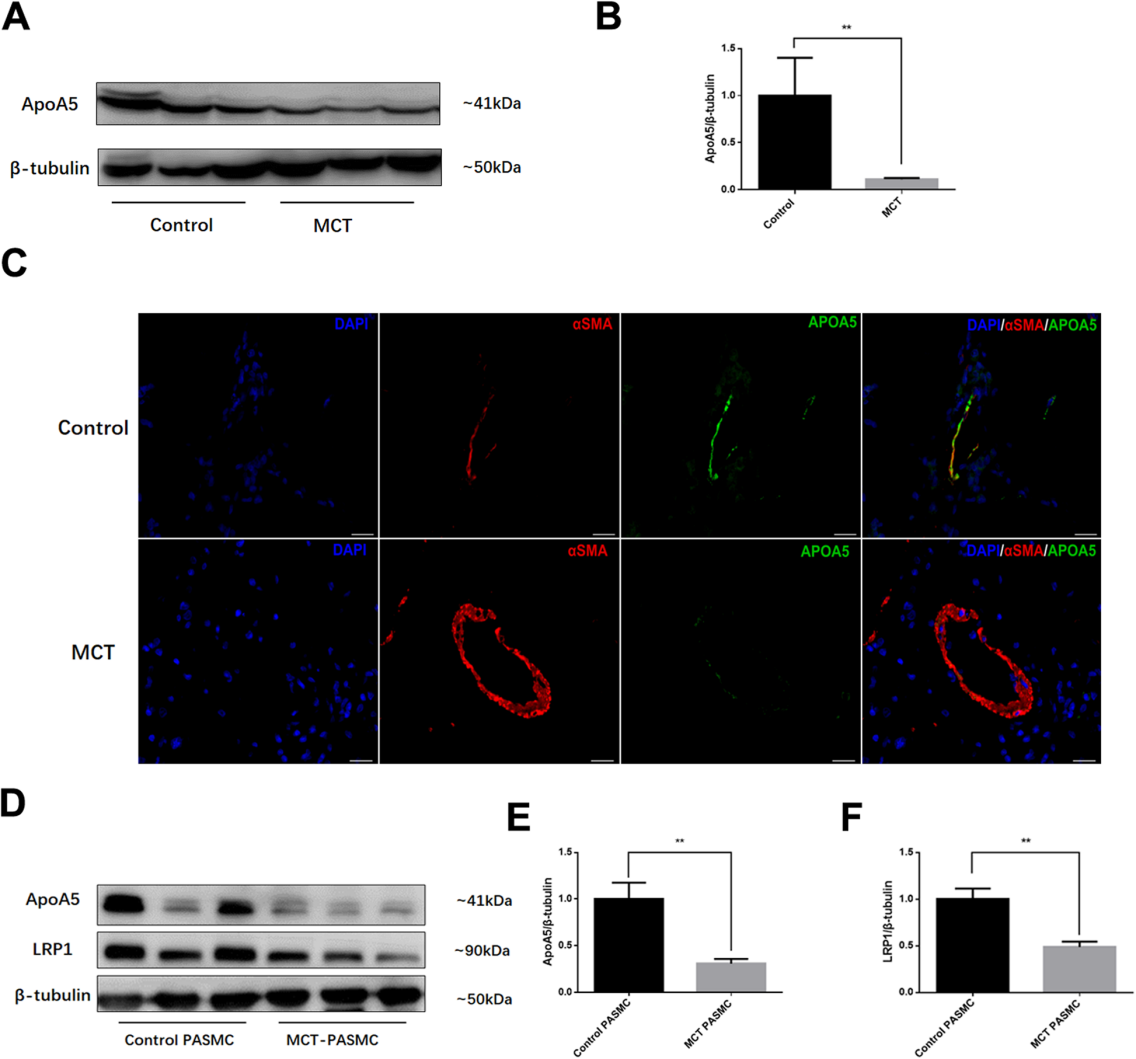
Decreased ApoA5 in pulmonary artery smooth muscle of MCT induced PAH at week 4. **A**, **B** Represent Western blot (**A**) and analysis (**B**) of lung ApoA5 expression in Control and MCT animals. (**C**)Co-localization of ApoA5 and pulmonary artery smooth muscle. (**D**-**F**) Represent western blot (D) and analysis (E, F) of ApoA5 and LRP1 expression in Control and MCT PASMCs. Data presented as Mean ± SD. For IF staining, scale bar is 25 μm. **, *P* < 0.01

Decreased ApoA5 was previously found in the serum of PAH patients as well as in the circulation and RV of the MCT-induced PAH animal model [12]. Here, the results showed that ApoA5 was also decreased in the lungs of MCT-treated PAH animals at week 4 (Fig. 1A, B). ApoA5 is a protein that is mostly synthesized in the liver and then secreted to the circulation; thus, ApoA5 expression in the liver was measured, and decreased ApoA5 expression was found in PAH rats (Supplemental Fig. 1A, B). The real-time PCR results also revealed that ApoA5 gene expression was decreased along with the expression of its modulating effectors cAMP response element-binding protein H (CREBH) and sterol regulatory element-binding protein (SREBP)-1C. Meanwhile, other 2 transcription factors, liver X receptor alpha (LXRα) and peroxisome proliferator activated receptor alpha (PPARα) haven’t showed any differences between these two groups (Supplemental Fig. 1C). Immunofluorescence staining showed that ApoA5 colocalized with pulmonary smooth muscle and was decreased in small arteries in MCT group (Fig. 1C). Considering that very low gene expression is observed in the lung and pulmonary vessels, ApoA5 is likely taken up from the circulation through low density lipoprotein receptor-related protein-1 (LRP1). Furthermore, PASMCs were isolated from the control and MCT-induced PAH rats, and the results showed decreased ApoA5 and LRP1 in the MCT group (Fig. 1D-F), thus verifying the reduced uptake of ApoA5 in PASMCs in MCT-induced PAH.

### Overexpression of ApoA5 ameliorates MCT-induced pulmonary artery remodeling

**Fig. 2:**
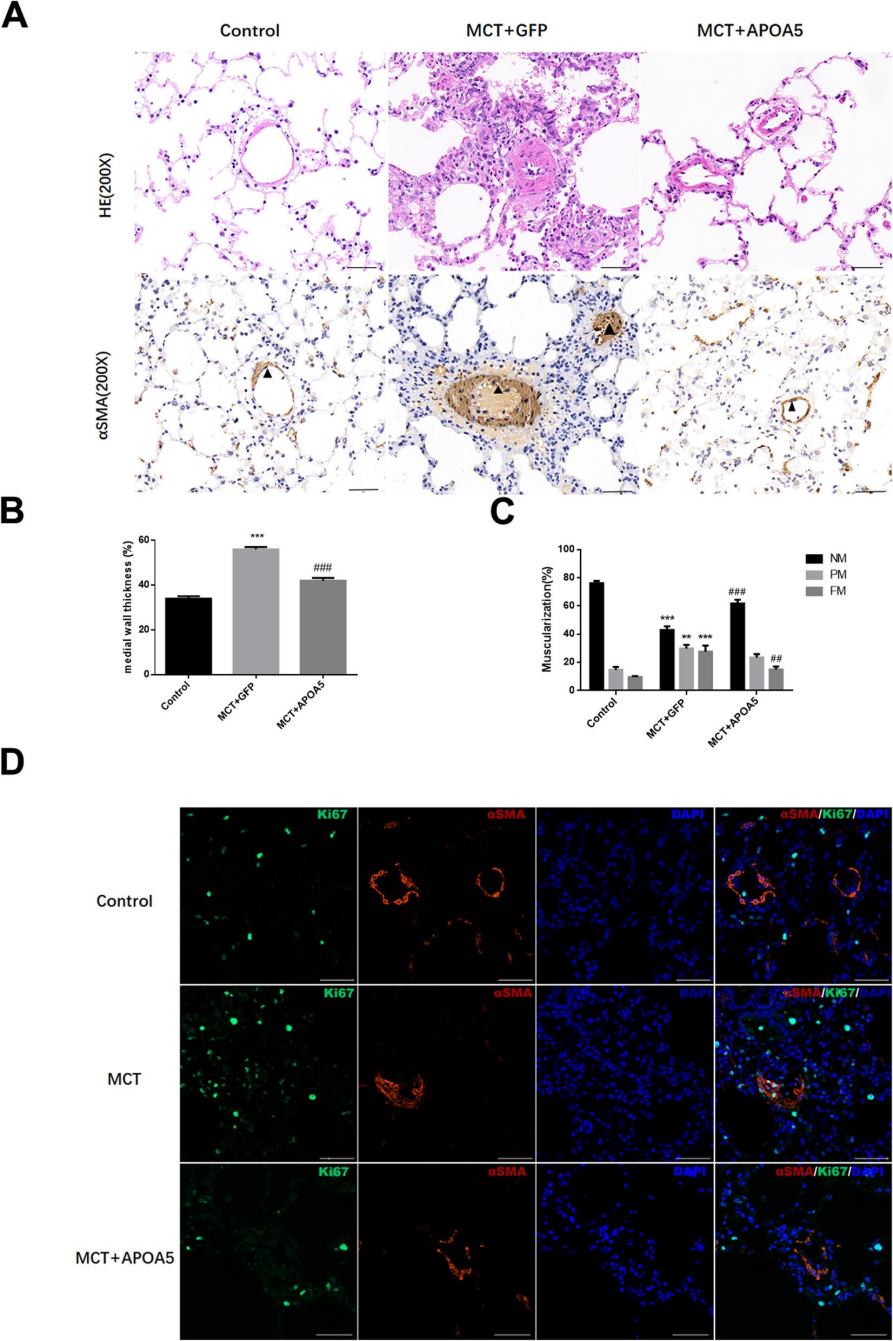
ApoA5 overexpression inhibiting pulmonary artery remodeling. **A** H&E staining and immunohistochemical staining of α-smooth muscle actin (α-SMA) showed ApoA5 inhibit the pulmonary artery remodeling. **B**, **C**) ApoA5 significantly alleviates MCT induced medial wall thickness and muscularization of small arteries. (**B**) Quantification of medial wall thickness; (**C**) Quantification of vessel muscularization; (**D**) Representative IF staining for Ki67 and α-SMA. For H&E, IHC and IF staining, Scale bar = 50 μm; For medial wall thickness and muscularization quantification, data presented as mean ± SEM, *n* = 3–4 for all groups. Only vessels with diameter between 30 and 100 μm were analyzed. NM, non-muscularized vessels; PM, partial muscularized vessels; FM, full muscularized vessels. ** Compared with Control group, *P* < 0.01; *** Compared with Control group, *P* < 0.001; ## Compared with MCT + GFP group, *P* < 0.01; ### Compared with MCT + GFP group, *P* < 0.001

Our previous work found that overexpressing ApoA5 had a protective effect on MCT-induced PAH and right heart failure [12]. Beyond its protective impact on right heart failure and RV fibrosis, ApoA5 could also ameliorate the thickening and proliferation of pulmonary artery smooth muscle. IF staining showed restored ApoA5 expression in pulmonary smooth muscle in MCT + ApoA5 group (Supplemental Fig. 2). Increased medial thickening was observed in the MCT-induced PAH group compared with the control group, while ApoA5 overexpression inhibited medial wall thickening (Fig. 2A, B). MCT increased the partial and full muscularization of the vessels, and the MCT + ApoA5 group showed a decrease in full muscularization (Fig. 2A, C). By Ki67 staining, MCT + ApoA5 group showed a decreased Ki67 in pulmonary vasculature that colocalized with α-SMA (Fig. 2D).

### ApoA5 overexpression inhibits PDGF-BB-induced PASMC proliferation

**Fig. 3:**
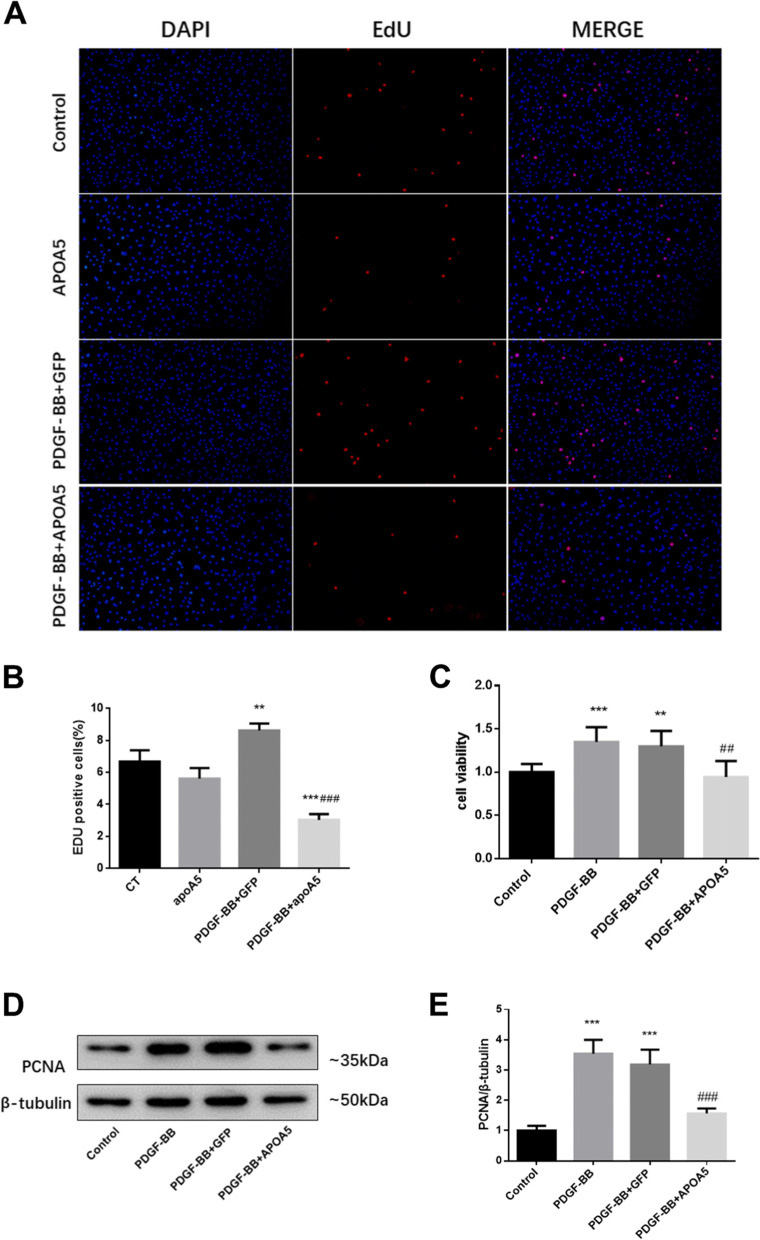
ApoA5 overexpression inhibit PDGF-BB induced PASMC proliferation. (**A**-**C**) EdU incorporation assay (**A**, **B**) and CCK8 (**C**) assay showed ApoA5 inhibits PDGF-BB induced PASMC proliferation. (**D**, **E**) Represent western blots and quantification (**E**) showing ApoA5 decreases expression of PCNA in PASMCs. All data shown as Mean ± SD; ** *P* < 0.01 compared to Control group; *** *P* < 0.001 compared to Control group; ## *P* < 0.01 compared to PDGF-BB or PDGF-BB + GFP groups; ### *P* < 0.001 compared to PDGF-BB or PDGF-BB + GFP group

Hyperplasia of PASMC is a very important feature of PAH and will leads to pulmonary vessel remodeling and occlusion [16]. Because ApoA5 was found to colocalize with α-SMA in vivo and inhibit smooth muscle hypertrophy and proliferation, further investigations focused on its role in PASMCs and protective mechanisms. PDGF-BB is a cytokine that highly increased in PAH and induces proliferation and migration of PASMC [17]. The CCK-8 and EdU incorporation assay results confirmed that ApoA5 inhibits PDGF-BB-induced PASMC proliferation (Fig. 3A-C). Western blots also showed that ApoA5 could inhibit the proliferation marker PCNA (Fig. 3D, E).

### ApoA5 functions through inhibition of endoplasmic reticulum stress

**Fig. 4:**
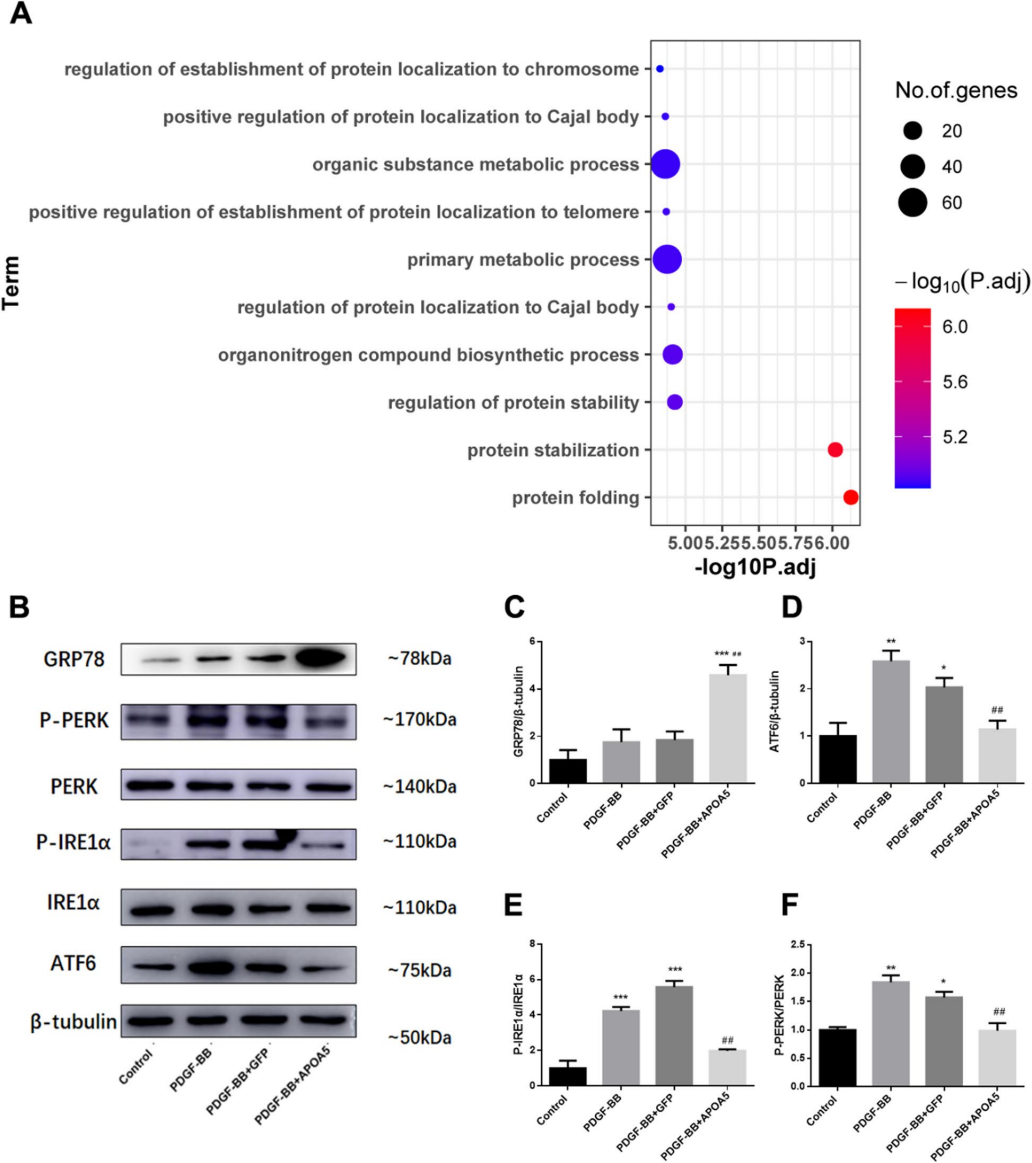
ApoA5 inhibits PDGF-BB induced PASMC proliferation by reducing ER stress. **A** Enriched Gene Ontology (GO) analysis showing the co-immunoprecipitation proteins that interacts with ApoA5; **B**-**F** Represent western blots **B** and quantification (**C**-**F**) showing the proteins that represent the ER stress status of the PASMC. All data presented as Mean ± SD. * *P* < 0.05 compared with control group; ** *P* < 0.01 compared with control group; *** *P* < 0.001 compared with control group; ## *P* < 0.01 compared with PDGF-BB or PDGF-BB + GFP group

**Fig. 5:**
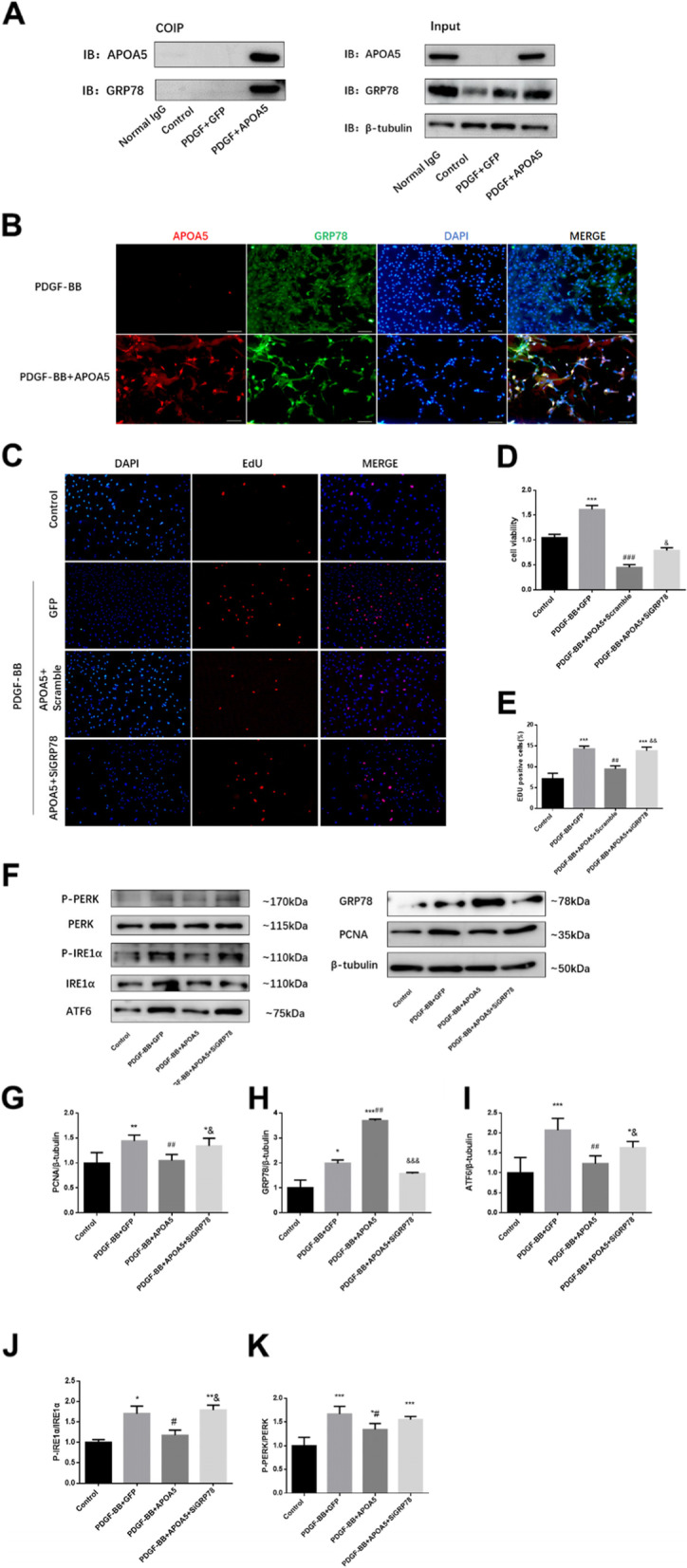
ApoA5 interacts with GRP78 to reduce PDGF-BB induced PASMC proliferation and ER stress. **A**, **B** co-IP **A** and immunofluorescence **B** showing the interacts between ApoA5 and GRP78. **C**-**E** Represent staining **C** and quantification E of EdU incorporation assay and CCK8 assay **D** showing that blocking the GRP78 by siRNA reversed the proliferation inhibition effect of ApoA5. (**F**) Represented western blot and quantification **G**-**K**) showing blocking the GRP78 by siRNA reversed effect of ApoA5 on ER stress. All data presented as Mean ± SD. For IF staining, Scale bar = 10 μm; *** *P* < 0.001 compared with control group; ** *P* < 0.01 compared with control group; * *P* < 0.05 compared with control group; ### *P* < 0.001 compared with PDGF-BB + GFP group; ## *P* < 0.01 compared with PDGF-BB + GFP group; # *P* < 0.05 compared with PDGF-BB + GFP group; &&& *P* < 0.001 compared with PDGF-BB + APOA5 + Scramble group; && *P* < 0.01 compared with PDGF-BB + APOA5 + Scramble group; & *P* < 0.05 compared with PDGF-BB + APOA5 + Scramble group

Since most works on ApoA5 have focused on lipid metabolism, to determine the mechanism underlying its ability to inhibit proliferation, the protein–protein interactions of ApoA5 were assessed using co-IP-MS. The results showed that proteins that interacted with ApoA5 were enriched in the protein stabilization and protein folding pathways (Fig. 4A). The ER mainly functions in protein folding, and ER stress is an important pathologic factor in pulmonary hypertension. To assess the role of ApoA5 in ER stress, the ER stress sensor GRP78 and its downstream ER stress activating pathways were tested. ApoA5 led to a sharp increase in GRP78, thus helping to inactivate ER stress pathways (Fig. 4B-F). Furthermore, immunofluorescence and co-IP assays showed that ApoA5 also colocalizes and interacts with GRP78, thus indicating a very complicated working mechanism between ApoA5 and GRP78 (Fig. 5A, B). Consequently, ApoA5 showed a protective effect on PDGF-BB-induced ER stress signaling. P-PERK, P-IRE1α, and ATF6 all returned to normal after the ApoA5 treatment (Fig. 4B-F).

### ApoA5 inhibits PASMC proliferation and ER stress by increasing GRP78 expression

To ensure that GRP78 leads to the proliferation inhibition effect of ApoA5, siRNA was used to knock down GRP78 to determine whether it blocked the impact of ApoA5. The EdU incorporation and CCK8 assays showed that ApoA5 blocked PASMC proliferation triggered by PDGF-BB. However, after GRP78 knockdown, the effect of ApoA5 was abrogated (Fig. 5C-E). Moreover, interfering with GRP78 also increased ER stress signaling, which corresponded to the cell proliferation status (Fig. 5F-K).

## Discussion

The etiology of pulmonary hypertension is complex, but common features include intimal and media hypertrophy, small vessels muscularization, stenosis and plexiform formation. These phenotypes are largely due to the high proliferation of vascular cells. Hyperplasia of PASMC is a very important feature of PAH and will leads to pulmonary vessel remodeling and occlusion [18]. Previous findings showed that ApoA5 protects against MCT-induced PAH and ameliorates right heart failure, and this study found that in addition to inhibiting RV fibrosis, ApoA5 has a protective effect on decreasing pulmonary pressure by inhibiting pulmonary vascular remodeling. These observations demonstrated that ApoA5 participated in the pathophysiologic change in PAH lung vasculature. Although right heart failure is the main reason for mortality in PAH, the current treatment strategies for PAH are mainly associated with vasodilation. The progression of pulmonary vasculature is still the biggest challenge [19, 20].

**Fig. 6:**
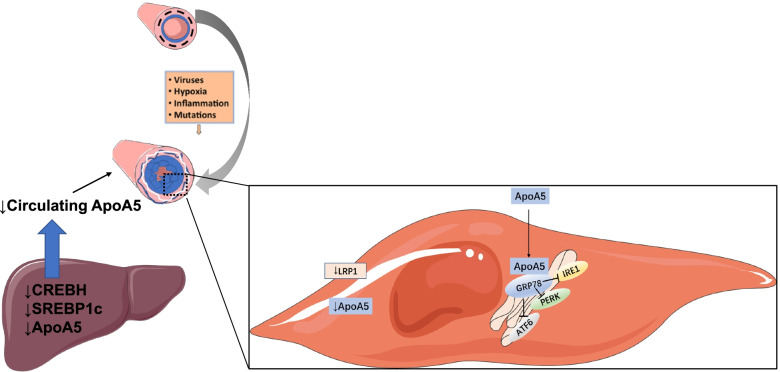
Summarization of the study. In MCT induced PAH animal model, decreased liver synthesis and PASMC uptake of ApoA5. Overexpress ApoA5 by adenovirus help to inhibit the PASMC proliferation by inhibiting ER stress pathways in a GRP78 dependent mechanism

This study found that the uptake of apoA5 in PASMCs of MCT induced PAH rats was significantly decreased. In addition, overexpression of apoA5 can reduce the thickness of medial wall and PASMC proliferation in MCT induced PAH model. To further explore the possible role of apoA5 in PASMCs, proteins interacting with apoA5 were measured by using coIP-MS, and found that most of them were enriched in pathways related to protein stability and protein folding (Fig. 6).

The ER is one of the most important organelles in cells and functions in protein synthesis, folding, transportation, and modification. Thus, it is highly sensitive to environmental homeostasis and cell stress [21]. Misfolded proteins in the ER can affect several cell functions and processes, including inflammation, differentiation, the cell cycle, and apoptosis [22, 23]. Moreover, the ER connects and communicates with several other organelles, especially mitochondria [24]. ER stress has been detected in PAH patients and many PAH animal models [25, 26]. Several studies have proven that both endogenous and exogenous ER stress inhibitors help protect against PAH and inhibit pulmonary artery remodeling and PASMC proliferation [27, 28]. Studies have confirmed that apoA5 is synthesized, located and secreted by ER [9]. Based on the above information, we speculated that apoA5 might inhibit the proliferation of pulmonary smooth muscle by improving ER stress.

ApoA5 has mainly been studied to identify its role in controlling serum TG metabolism, energy metabolism, and insulin sensitivity as a lipoprotein, and it has been shown to decrease lipid accumulation in cardiomyocytes [10]. Although a correlation between ApoA5 gene polymorphisms and cardiovascular diseases, such as hypertension, myocardial infarction, or stroke has been showed, previous studies have not focused on the underlying mechanism among blood vessels [29]. This study is the first to show that ApoA5 is decreased in PAH and elucidate its protective role against PAH. Although ApoA5 is only synthesized in the liver, it is found in many organs, such as adipocyte tissue and the heart, through receptor-mediated uptake [8, 10]. In MCT-induced PAH model, we detected that ApoA5 and its receptor LRP1 were decreased in PAH PASMCs. Meanwhile, the synthesis of ApoA5 is reduced in the liver of PAH animals. In this study, in vivo experiments indicated that ApoA5 could be taken up by the pulmonary artery and prevent its remodeling. Further in vitro experiments confirmed that ApoA5 could inhibit PASMC proliferation and modulate the ER stress sensor protein GRP78. GRP78 is a sensor protein of endoplasmic reticulum stress, and its expression level is increased during the onset of endoplasmic reticulum stress [30]. It binds to the relevant endoplasmic reticulum stress signaling proteins ATF6, PERK, and IRE1, and inhibits their activation [22]. As we observed a sharp increase of GRP78 and inhibition of ER stress, we speculate that, the effects of ApoA5 is induced by GRP78. After blocking GRP78, the protective effect of ApoA5 was abolished (Fig. 6).

Although not fully investigated, PAH is a disease related to many triggers. The MCT-induced PAH animal model is characterized by acute and severe PAH, inflammation, and lung fibrosis [31]. ER stress also participates in lung inflammation and fibrosis, which might be another reason for the protective effect of ApoA5. A previous study found that ApoA5 has an important impact on inhibiting LPS-induced acute liver inflammation through NF-KB signaling, which should be another important pathway that participates in MCT-induced PAH [14]. Moreover, ApoA5 is the most induced protein in liver regeneration, which indicates that ApoA5 might have a role in modulating cell proliferation or the cell cycle [32].

### Study strengths and limitations

The present study showed that ApoA5 helped improve vasculature remodeling as well as abnormal PASMC proliferation by inhibiting ER stress. This is the first study to reveal the role and detailed mechanism of ApoA5 in vascular disease. However, several limitations were observed in our study. First, we only proved the protective effect of ApoA5 in the MCT-induced PAH model. Beyond the MCT-induced model, ER stress has been confirmed in PAH patients and several other PAH animal models, and the effect of ApoA5 on these models should be investigated. Second, this is a preliminary study focused on investigating the mechanism of ApoA5 in inhibiting PASMC proliferation. We used adenovirus-induced overexpression, which might not be a good method for translational research. Further studies using recombinant protein or mimic peptides need to be performed.

## Conclusions

In summary, our study demonstrated that ApoA5 protected against MCT-induced PAH and PASMC proliferation by inhibiting GRP78-induced ER stress. These findings revealed the therapeutic role of ApoA5 and suggested that it could be a novel target in PAH.

## Supplementary Information

**Additional file 1: Supplemental Figure 1.** Decreased ApoA5 synthesis in MCT induced PH animal liver. (A, B) Represent western blot (A) and densitometry (B) of decreased liver ApoA5 synthesis in MCT induced PH rats. (C) Decreased transcript factor genes that modulating ApoA5 synthesis. *,*P*<0.05; ***, *P*<0.001. **Supplemental Figure 2.** Representative photographs of immunofluorescence staining for ApoA5 after overexpression. Scale bar is 25μm.

## Data Availability

All data related to this study are available upon request.

## Abbreviations

ApoA5: Apolipoprotein A5
PAH: Pulmonary arterial hypertension
PASMC: Pulmonary artery smooth muscle cell
TG: Triglyceride
MCT: Monocrotaline
GRP78: Glucose-regulated protein 78
PDGF-BB: Platelet growth factor-BB
FBS: Fetal bovine serum
COIP-MS: Coimmunoprecipitation-mass spectrometry
LRP1: LDL receptor-related protein-1
PERK: Double-stranded RNA-activated protein kinase (PKR)-like endoplasmic reticulum kinase
IRE1α: Inositol requiring 1 alpha
ATF6: Activating transcription factor 6

## References

[1] Tuder RM, Archer SL, Dorfmüller P, Erzurum SC, Guignabert C, Michelakis E, Rabinovitch M, Schermuly R, Stenmark KR, Morrell NW (2013). Relevant issues in the pathology and pathobiology of pulmonary hypertension. J Am College of Cardiol.

[2] Shimoda LA, Laurie SS (2013). Vascular remodeling in pulmonary hypertension. J Mol Med.

[3] Jeffery TK, Wanstall JC (2001). Pulmonary vascular remodeling: a target for therapeutic intervention in pulmonary hypertension. Pharmacol Ther.

[4] Wang A, Valdez-Jasso D (2021). Cellular mechanosignaling in pulmonary arterial hypertension. Biophys Rev.

[5] Vaillancourt M, Ruffenach G, Meloche J, Bonnet S (2015). Adaptation and remodelling of the pulmonary circulation in pulmonary hypertension. Can J Cardiol.

[6] Humbert M, Guignabert C, Bonnet S, Dorfmüller P, Klinger JR, Nicolls MR, Olschewski AJ, Pullamsetti SS, Schermuly RT, Stenmark KR (2019). Pathology and pathobiology of pulmonary hypertension: state of the art and research perspectives. Eur Respir J.

[7] Schaap FG, Rensen PC, Voshol PJ, Vrins C, van der Vliet HN, Chamuleau RA, Havekes LM, Groen AK, van Dijk KW (2004). ApoAV reduces plasma triglycerides by inhibiting very low density lipoprotein-triglyceride (VLDL-TG) production and stimulating lipoprotein lipase-mediated VLDL-TG hydrolysis. J Biol Chem.

[8] Zheng X-Y, Zhao S-P, Yu B-L, Wu C-L, Liu L (2012). Apolipoprotein A5 internalized by human adipocytes modulates cellular triglyceride content. Biol Chem.

[9] Forte TM, Ryan RO (2015). Apolipoprotein A5: extracellular and intracellular roles in triglyceride metabolism. Curr Drug Targets.

[10] Luo J, Xu L, Li J, Zhao S (2018). Effects and mechanisms of apolipoprotein AV on the regulation of lipid accumulation in cardiomyocytes. Lipids Health Dis.

[11] Brittain EL, Talati M, Fessel JP, Zhu H, Penner N, Calcutt MW, West JD, Funke M, Lewis GD, Gerszten RE (2016). Fatty acid metabolic defects and right ventricular lipotoxicity in human pulmonary arterial hypertension. Circulation.

[12] Chen J, Luo J, Yang X (2021). Expression of ApoA5 and its function in the right ventricular failing and remodeling secondary to pulmonary hypertension[J]. J Cell Physiol..

[13] Lin Y-C, Nunez V, Johns R, Shiao SPK (2017). APOA5 gene polymorphisms and cardiovascular diseases: metaprediction in global populations. Nurs Res.

[14] Tao YC, Wang ML, Wu DB, Luo C, Tang H, Chen EQ (2019). Apolipoprotein A5 alleviates LPS/D-GalN-induced fulminant liver failure in mice by inhibiting TLR4-mediated NF-kappaB pathway. J Transl Med.

[15] Wang C, Cui Y, Miao H, Xiong X, Dou J, Shao L, Tang X, Zhang Y (2020). Apolipoprotein A-V Is a Novel Diagnostic and Prognostic Predictor in Pediatric Patients with Sepsis: A Prospective Pilot Study in PICU. Mediators Inflamm.

[16] Houssaini A, Abid S, Mouraret N, Wan F, Rideau D, Saker M, Marcos E, Tissot C-M, Dubois-Randé J-L, Amsellem V (2013). Rapamycin reverses pulmonary artery smooth muscle cell proliferation in pulmonary hypertension. Am J Respir Cell Mol Biol.

[17] Zhao Y, Lv W, Piao H, Chu X, Wang H (2014). Role of platelet-derived growth factor-BB (PDGF-BB) in human pulmonary artery smooth muscle cell proliferation. J Recept Signal Transduct Res.

[18] Mandegar M (2004). Fung Y-CB, Huang W, Remillard CV, Rubin LJ, Yuan JX-J: cellular and molecular mechanisms of pulmonary vascular remodeling: role in the development of pulmonary hypertension. Microvasc Res.

[19] Crnkovic S, Egemnazarov B, Damico R, Marsh LM, Nagy BM, Douschan P, Atsina K, Kolb TM, Mathai SC, Hooper JE (2019). Disconnect between fibrotic response and right ventricular dysfunction. Am J Respir Crit Care Med.

[20] Voelkel NF, Quaife RA, Leinwand LA, Barst RJ, McGoon MD, Meldrum DR, Dupuis J, Long CS, Rubin LJ, Smart FW (2006). Right ventricular function and failure: report of a National Heart, Lung, and Blood Institute working group on cellular and molecular mechanisms of right heart failure. Circulation.

[21] Schröder M, Kaufman RJ (2005). ER stress and the unfolded protein response. Mutat Res.

[22] Hetz C (2012). The unfolded protein response: controlling cell fate decisions under ER stress and beyond. Nat Rev Mol Cell Biol.

[23] Hetz C, Papa FR (2018). The unfolded protein response and cell fate control. Mol Cell.

[24] Lopez-Crisosto C, Pennanen C, Vasquez-Trincado C, Morales PE, Bravo-Sagua R, Quest AF, Chiong M, Lavandero S (2017). Sarcoplasmic reticulum–mitochondria communication in cardiovascular pathophysiology. Nat Rev Cardiol.

[25] Wang J-J, Zuo X-R, Xu J, Zhou J-Y, Kong H, Zeng X-N, Xie W-P, Cao Q (2016). Evaluation and treatment of endoplasmic reticulum (ER) stress in right ventricular dysfunction during monocrotaline-induced rat pulmonary arterial hypertension. Cardiovasc Drugs Ther.

[26] Cao X, He Y, Li X, Xu Y, Liu X (2019). The IRE1α-XBP1 pathway function in hypoxia-induced pulmonary vascular remodeling, is upregulated by quercetin, inhibits apoptosis and partially reverses the effect of quercetin in PASMCs. Am J Transl Res.

[27] Wu Y, Adi D, Long M, Wang J, Liu F, Gai M-T, Aierken A, Li M-Y, Li Q, Wu L-Q (2016). 4-Phenylbutyric acid induces protection against pulmonary arterial hypertension in rats. PLoS One.

[28] Dromparis P, Paulin R, Stenson TH, Haromy A, Sutendra G, Michelakis ED (2013). Attenuating endoplasmic reticulum stress as a novel therapeutic strategy in pulmonary hypertension. Circulation.

[29] Guardiola M, Ribalta J (2017). Update on APOA5 genetics: toward a better understanding of its physiological impact. Curr Atheroscler Rep.

[30] Sano R, Reed JC (2013). ER stress-induced cell death mechanisms. Biochim Biophys Acta..

[31] Wilson DW, Segall H, Pan L, Lame M, Estep J, Morin D (1992). Mechanisms and pathology of monocrotaline pulmonary toxicity. Crit Rev Toxicol.

[32] van der Vliet HN, Sammels MG, Leegwater AC, Levels JH, Reitsma PH, Boers W, Chamuleau RA (2001). Apolipoprotein A-V: a novel apolipoprotein associated with an early phase of liver regeneration. J Biol Chem.

