# Supplementary material for: Apolipoprotein A5 ameliorates MCT induced pulmonary hypertension by inhibiting ER stress in a GRP78 dependent mechanism
Source: Lipids Health Dis. 2022 Aug 8;21:69. doi: 10.1186/s12944-022-01680-4 (PMC9358849; doi:10.1186/s12944-022-01680-4)
Supplement: Supplementary file 1 — Additional file 1: Supplemental Figure 1. Decreased ApoA5 synthesis in MCT induced PH animal liver. (A, B) Represent western blot (A) and densitometry (B) of decreased liver ApoA5 synthesis in MCT induced PH rats. (C) Decreased transcript factor genes that modulating ApoA5 synthesis. *,P<0.05; ***, P<0.001. Supplemental Figure 2. Representative photographs of immunofluorescence staining for ApoA5 after overexpression. Scale bar is 25μm. [file 12944_2022_1680_MOESM1_ESM.docx]

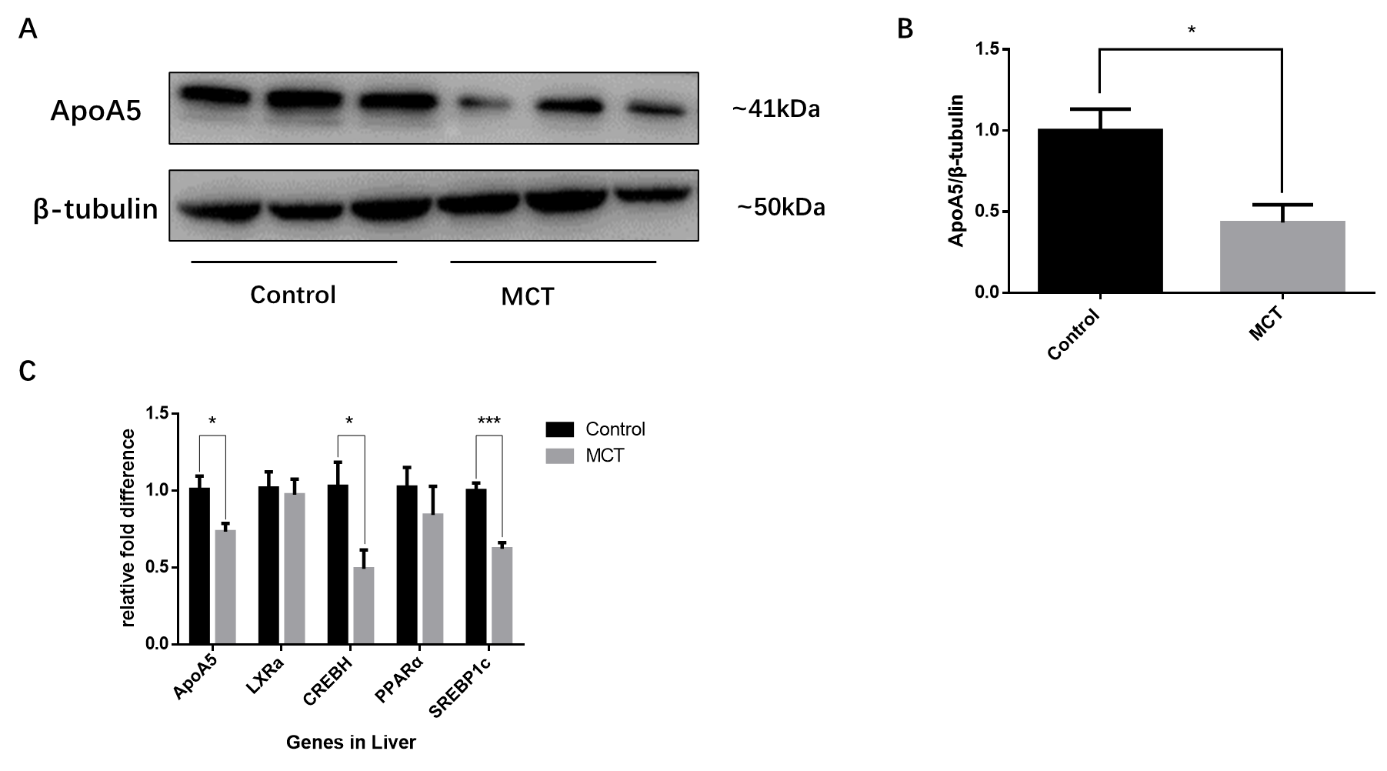


Supplemental Figure 1. Decreased ApoA5 synthesis in MCT induced PH animal liver.

(A, B) Represent western blot (A) and densitometry (B) of decreased liver ApoA5 synthesis in MCT induced PH rats. (C) Decreased transcript factor genes that modulating ApoA5 synthesis. *,*P*<0.05; ***, *P*<0.001.


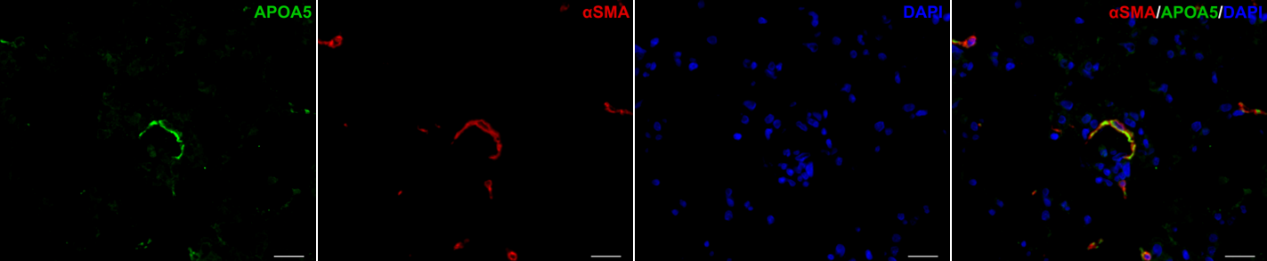


Supplemental Figure 2. Representative photographs of immunofluorescence staining for ApoA5 after overexpression

Scale bar is 25μm.
